# Coronary Plaque Erosion: Epidemiology, Diagnosis, and Treatment

**DOI:** 10.3390/ijms25115786

**Published:** 2024-05-26

**Authors:** Panagiotis Theofilis, Panayotis K. Vlachakis, Aggelos Papanikolaou, Paschalis Karakasis, Evangelos Oikonomou, Konstantinos Tsioufis, Dimitris Tousoulis

**Affiliations:** 1 1st Department of Cardiology, “Hippokration” General Hospital, National and Kapodistrian University of Athens, 11527 Athens, Greece; panos.theofilis@hotmail.com (P.T.); vlachakispanag@gmail.com (P.K.V.); agepap25@otenet.gr (A.P.); kptsioufis@gmail.com (K.T.); 2 2nd Department of Cardiology, “Hippokration” General Hospital, Aristotle University of Thessaloniki, 54642 Thessaloniki, Greece; pakar15@hotmail.com; 3 3rd Department of Cardiology, Thoracic Diseases General Hospital “Sotiria”, National and Kapodistrian University of Athens, 11527 Athens, Greece; boikono@gmail.com

**Keywords:** atherosclerosis, coronary artery disease, acute coronary syndrome, plaque erosion, molecular mechanisms, optical coherence tomography

## Abstract

Plaque erosion (PE), a distinct etiology of acute coronary syndromes (ACSs), is often overshadowed by plaque ruptures (PRs). Concerning its epidemiology, PE has garnered increasing recognition, with recent studies revealing its prevalence to be approximately 40% among ACS patients, challenging earlier assumptions based on autopsy data. Notably, PE exhibits distinct epidemiological features, preferentially affecting younger demographics, particularly women, and often manifesting as a non-ST-segment elevation myocardial infarction. There are seasonal variations, with PE events being less common in winter, potentially linked to physiological changes and cholesterol solidification, while peaking in summer, warranting further investigation. Moving to molecular mechanisms, PE presents a unique profile characterized by a lesser degree of inflammation compared to PR, with endothelial shear stress emerging as a plausible molecular mechanism. Neutrophil activation, toll-like receptor-2 pathways, and hyaluronidase 2 expression are among the factors implicated in PE pathophysiology, underscoring its multifactorial nature. Advancements in intravascular imaging diagnostics, particularly optical coherence tomography and near-infrared spectroscopy coupled with intravascular ultrasound, offer unprecedented insights into plaque composition and morphology. Artificial intelligence algorithms show promise in enhancing diagnostic accuracy and streamlining image interpretation, augmenting clinician decision-making. Therapeutically, the management of PE evolves, with studies exploring less invasive approaches such as antithrombotic therapy without stenting, particularly in cases identified early through intravascular imaging. Additionally, the potential role of drug-coated balloons in reducing thrombus burden and minimizing future major adverse cardiovascular events warrants further investigation. Looking ahead, the integration of advanced imaging modalities, biomarkers, and artificial intelligence promises to revolutionize the diagnosis and treatment of coronary PE, ushering in a new era of personalized and precise cardiovascular care.

## 1. Introduction

Acute coronary syndromes (ACSs) constitute a significant burden on global healthcare systems and remain a leading cause of morbidity and mortality worldwide. Within the spectrum of ACS, plaque rupture (PR) has traditionally garnered considerable attention as the predominant etiology, shaping therapeutic strategies and research agendas. The management of ACS has evolved with the introduction of newer antiplatelet agents, more potent statins, and novel therapies targeting inflammation and plaque stabilization. Additionally, early invasive strategies like coronary angiography and percutaneous coronary intervention (PCI) have become the standard of care.

Although the existing therapeutic advances in this field have been based on the concept of ruptured plaques, recent insights have illuminated the role of another distinct culprit: plaque erosion (PE). While often overshadowed by PR, PE represents a substantial portion of ACS cases, with its epidemiology, diagnosis, and management offering a rich terrain for exploration and innovation. Advances in intravascular imaging techniques have improved our ability to detect and characterize PE. Interestingly, such precise plaque characterization might have therapeutic implications, with personalized approaches to PE management. Therefore, in this review article, we delve into the multifaceted landscape of coronary PE, aiming to provide a comprehensive overview of its epidemiology, diagnostic modalities, and therapeutic considerations.

## 2. Plaque Phenotypes

### 2.1. Plaque Rupture

Coronary thrombosis driven by PR constitutes a significant portion, approximately 75%, of all ACSs. Various conventional cardiovascular risk factors like hypertension, smoking, diabetes, hyperlipidemia, and obesity, alongside non-traditional factors like inflammation, infection, and genetic predisposition, collectively contribute to the development of vulnerable plaques. For instance, hypertension imposes mechanical strain on the arterial wall, accelerating plaque formation and elevating the likelihood of rupture. Diabetes and smoking exacerbate plaque inflammation, compromise endothelial function, and foster plaque instability.

The inception of PR occurs within a vulnerable plaque, characterized by a thin fibrous cap overlaying a lipid-laden core [1]. Beneath this cap lies a cluster of inflammatory cells, including macrophages and T lymphocytes, actively involved in ongoing plaque inflammation and restructuring [1]. The breach of the fibrous cap exposes the lipid core to the bloodstream, initiating a cascade of events [1]. Mechanical stress, inflammation, and degradation of the extracellular matrix are among the factors precipitating PR. Hemodynamic factors like shear stress and cyclic strain exert mechanical strain on the plaque, leading to cap thinning and eventual rupture. Moreover, prolonged inflammation within the plaque prompts the release of proteolytic enzymes, particularly matrix metalloproteinases (MMPs), which degrade components of the extracellular matrix [2]. An imbalance between MMPs and their inhibitors weakens the fibrous cap, rendering it more susceptible to rupture [2]. Inflammation plays a pivotal role in both the initiation and progression of PR. Circulating monocytes adhere to activated endothelial cells, infiltrating into the intima where they differentiate into macrophages [3]. These macrophages, upon internalizing oxidized low-density lipoproteins, transform into foam cells, contributing to the formation of the lipid core [3]. Inflammatory cytokines such as interleukin-6 (IL-6) and tumor necrosis factor-alpha (TNF-α) heighten plaque vulnerability by perpetuating inflammation [4]. Additionally, local inflammatory mediators such as C-reactive protein (CRP) significantly contribute to plaque destabilization, elevating the risk of rupture [4].

### 2.2. Calcified Nodule

The role of calcified nodules in ACS has gained increasing attention. Calcified nodules refer to discrete calcified structures within the coronary arteries that can contribute to luminal narrowing and subsequent ischemic events. The formation of calcified nodules in coronary arteries involves a complex interplay of biological processes, including inflammation, osteogenic transformation, and matrix remodeling [5]. Chronic inflammation within atherosclerotic plaques triggers the release of pro-inflammatory cytokines and growth factors, stimulating the migration and differentiation of vascular smooth muscle cells (VSMCs) into osteoblast-like cells. These VSMCs promote the deposition of calcium phosphate crystals, leading to the development of calcified nodules. Matrix remodeling, characterized by altered expression of matrix metalloproteinases and their inhibitors, further contributes to the process. Calcified nodules are responsible for 5.3% of ACS [6]. Several traditional risk factors for atherosclerosis, including advanced age, male gender, hypertension, dyslipidemia, and diabetes mellitus, are associated with an increased likelihood of calcified nodules [7]. In addition, emerging risk factors such as chronic kidney disease and genetic predisposition have been implicated [7]. These risk factors promote vascular calcification by disrupting the balance between calcification inhibitors and promoters, favoring the deposition of calcium within the arterial wall.

### 2.3. Plaque Erosion

**Figure 1:**
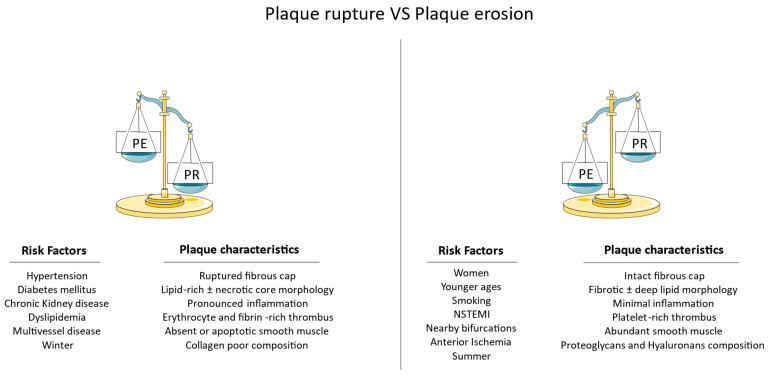
Risk factors and plaque characteristics in plaque erosion (PE) vs. plaque rupture (PR). NSTEMI—non-ST-segment elevation myocardial infarction.

While calcified nodules are characterized by the presence of focal calcifications within the atherosclerotic plaque and PR involves the rupture of an atherosclerotic plaque’s fibrous cap, PE predominantly involves superficial erosion of the denuded endothelium without extensive calcification (Figure 1). The presence of PE in culprit coronary arteries of patients with ACS has been increasingly more common over the years. Two main factors could be responsible for this observation. First and foremost, although early studies have noted a PE prevalence in approximately 20% of ACS patients, they were based on autopsy studies of fatal acute cardiovascular events that may be more commonly associated with PR [8]. Invasive vascular imaging was not widely used; thus, these rates could have been falsely low. Moreover, the increased awareness regarding the management of major cardiovascular risk factors may have accounted for the attenuation of atherosclerosis progression and the stabilization of atherosclerotic plaques [8]. Specifically, aggressive lipid-lowering therapies (statins, ezetimibe, bempedoic acid, and proprotein convertase subtilisin/kexin type 9 (PCSK9) inhibitors including inclisiran) [9,10,11,12,13], novel antidiabetic agents with pleiotropic effects (sodium-glucose cotransporter-2 inhibitors, glucagon-like peptide-1 receptor agonists) [14,15], and contemporary antihypertensive medications (renin-angiotensin-aldosterone system blockers) are common approaches with significant anti-atherosclerotic effects. Smoking cessation and adaptation of a healthier lifestyle in terms of diet and exercise could be essential determinants of atherosclerosis stabilization [16,17,18]. Τhe importance of inflammation should be also stressed, as the residual inflammatory risk is a crucial aspect of the atherosclerotic process that could be medically managed with agents such as colchicine [19], sodium-glucose cotransporter-2 inhibitors [20], and others. As a result, the regression of atherosclerosis could prevent vulnerable, rupture-prone plaque formations and lead to more cases of non-ST-segment elevation myocardial infarction (NSTEMI) with PE. We should also mention that the use of invasive vascular imaging methods and the establishment of possible PE criteria could have contributed to a greater degree of PE detection. It is, therefore, unsurprising that contemporary studies in the field have produced PE rates of approximately 40% [8]. However, even such rates may not reflect the true prevalence of PE, since only a limited proportion of patients with ACS undergo invasive coronary imaging with optical coherence tomography (OCT) or intravascular ultrasound (IVUS).

**Figure 2:**
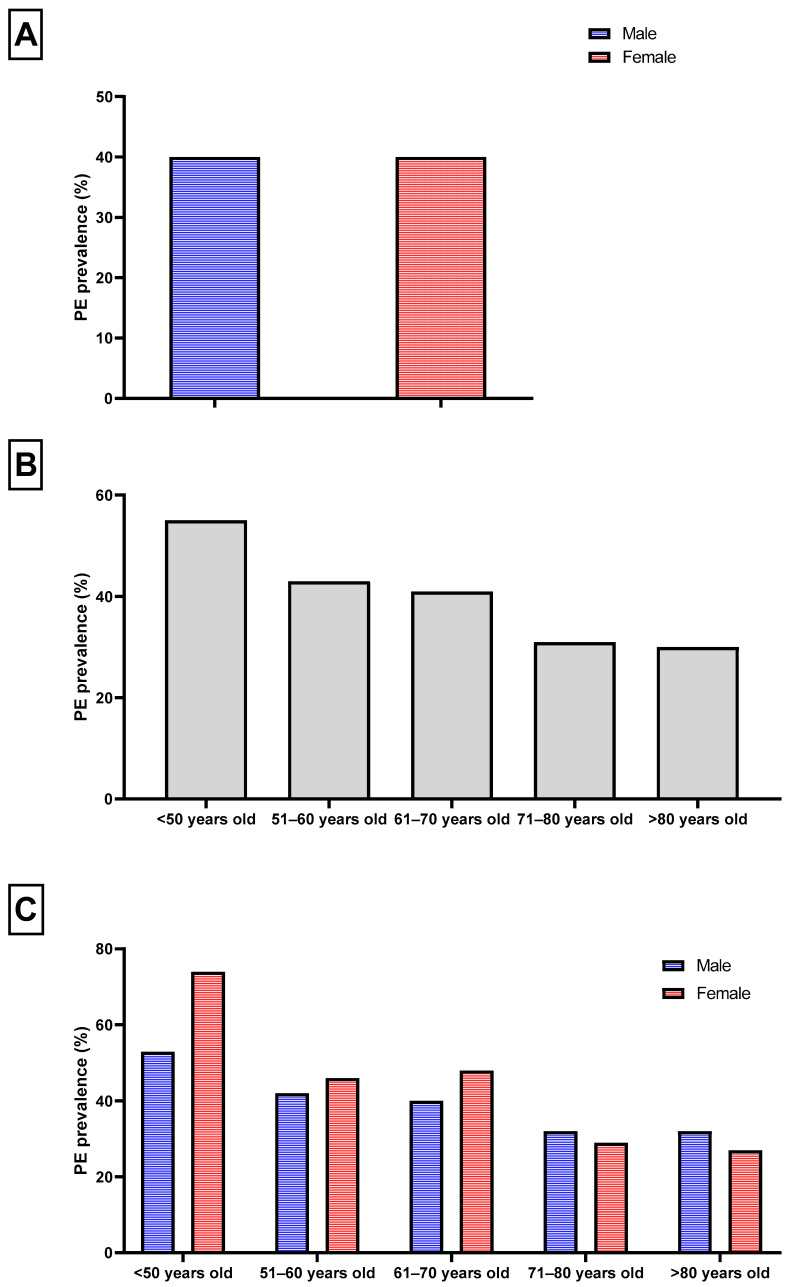
Trends in plaque erosion (PE) prevalence according to (**A**) sexes, (**B**) age categories, and (**C**) age-sex categories.

The epidemiology of PE has distinct characteristics. To begin with, it appears that men and women are equally affected overall, presenting with PE in 40% of ACS based on the study of Seegers et al. (Figure 2) [21]. When looking at the trends of PE prevalence according to age, a stepwise decrease is noted, with higher rates being recorded in those <70 years of age (<50: 55%, 51–60: 43%, 61–70: 41%, Figure 2). The evidence is lacking regarding racial differences in PE prevalence, with one study showing similar rates in Asian and White patients after adjustment for confounders [22].

Concerning additional age-related characteristics of patients with ACS caused by PE, younger patients present more commonly with thrombus (and a STEMI), while the elderly more frequently have greater percentage diameter stenoses and lipid-rich and calcified plaques [23]. In an attempt to better characterize PE prevalence and risk factors solely in women with ACS, Seegers et al. performed OCT prior to PCI in 382 women and found that PE was more common in younger women (<60 years old) and in NSTE-ACS [24]. Among the examined factors, a family history of CAD was predictive of PE presence [24]. Additionally, ACS events triggered by PE occur at lower rates during the winter season compared to the summer season (odds ratio (OR): 0.62, 95% confidence interval (CI): 0.43–0.91, *p* = 0.013). This interesting observation could potentially be explained by the increases in blood pressure at lower temperatures due to the stimulation of cold skin receptors and the rise in circulating catecholamines [25], together with cholesterol solidification [26], rendering a coronary plaque more prone to rupture. On the contrary, a greater incidence of PE during the summer season is a finding that mandates further research regarding its pathophysiologic basis.

Eroded plaques are less likely to cause STEMIs but are frequently implicated in cases of NSTEMI [27]. Several studies have found that PE is preferentially localized in the left anterior descending artery (LAD), notably in the proximal and mid segments, which are grouped around a bifurcation [28,29,30]. This usual distribution may represent distinct local hemodynamic pressures working within the coronary circulation, such as altered endothelial shear stress, which may be more prominent in a conduit that has side branches, such as the LAD [31]. In terms of angiographic properties, PE is associated with a less complicated and widespread atherosclerotic pattern than PR [28,29,30]. PE was associated with a lower prevalence of multivessel CAD (28.5% vs. 49.6%, *p* < 0.001), a lower Gensini score (21.3 vs. 25.6, *p* = 0.014), and a lower Syntax score (8.9 vs. 11.5, *p* < 0.001) compared to PR in a cohort of NSTE-ACS patients [28]. These characteristics are consistent with OCT evidence revealing decreased pancoronary vulnerability in patients with PE, and may help to explain why these individuals have a better prognosis than those with PR [32,33]. PE lesions are less commonly related with a baseline thrombolysis in myocardial infarction (TIMI) flow grade 1. Furthermore, PE is frequently linked with a simpler angiographic lesion phenotype. PE generally exhibits a more “simple” A/B1 lesion phenotype when using the ACC/AHA lesion categorization, as opposed to PR, which typically exhibits a “complex” B2/C lesion phenotype [34]. PE is more commonly linked with a concentric type I lesion, whereas PR is associated with an eccentric type II lesion, according to the Ambrose lesion classification [34]. PE, unlike PR, is less usually associated with angiographic signs of calcification and thrombus [34]. However, PE could be on a background of a lipid-rich plaque (maximal lipid arc >180°), which may share similarities with PR, such as the presentation (STEMI), peak creatine kinase level, and small dense low-density lipoprotein cholesterol levels of affected patients, as opposed to PE of a fibrous plaque [35].

Prognosis also differs between PR and PE. In an analysis of 398 consecutive ACS patients (62% PR, 25% PE), major adverse cardiovascular events (cardiac mortality, recurrent ACS, hospitalization for unstable angina, and target vessel revascularization) occurred less frequently in patients with PE (14.3% vs. 26.7%, *p* = 0.02) [36]. In another analysis of 702 ACS patients treated with OCT-guided PCI, the subgroup of patients with PE as the underlying pathophysiology faced the lowest proportion of major adverse cardiac events at 1 year when compared to PR and calcified nodules (6.2% vs. 12.4% vs. 32.1%, *p* < 0.0001) [37]. Specifically, patients with PR and calcified nodules have a 2.18-fold and 4.49-fold higher risk of 1-year major adverse cardiac events, respectively, with PE as the reference [37].

Last but not least, patients with PE frequently exhibit less extensive non-coronary atherosclerosis compared to PR as evidenced by peripheral plaque prevalence (79.1% vs. 93.4%, *p* < 0.001), as well as a lower number of peripheral atherosclerotic plaques [38]. Characteristics of peripheral lesion vulnerability (surface irregularity, plaque heterogeneity, and calcification) are also less commonly seen in patients with ACS caused by PE [38].

## 3. Molecular Mechanisms of PE

Histologically, eroded plaques exhibit distinct features that differentiate them from ruptured plaques. Eroded plaques often have a thick fibrous cap without evidence of cap disruption. Instead of a lipid core, they commonly demonstrate superficial platelet-rich (white) thrombi adherent to an intact endothelial surface. Eroded plaques were rich in extracellular matrix components, such as proteoglycans and glycosaminoglycans, rather than lipids. Eroded lesions had fewer inflammatory cells and more vascular smooth muscle cells than ruptured plaques. The absence of a fibrous cap rupture and the presence of adherent thrombi are key histopathological characteristics distinguishing PE from PR.

Regarding the molecular mechanisms implicated in PE pathophysiology, an inflammatory process of a lesser magnitude is described in PE compared to PR, as shown by a lower inflammatory expression of NLRP3 inflammasome downstream products such as IL-1β-associated proteins and IL-6 [36].

The role of endothelial sheer stress has been increasingly speculated as a molecular mechanism of PE development. Based on the study of Hakim et al. in patients who underwent coronary angiography and OCT examination, maximal endothelial sheer stress (OR: 1.32, 95% CI: 1.06–1.65, *p* = 0.014) and maximal endothelial sheer stress gradient in any direction (OR: 1.22, 95% CI: 1.03–1.45, *p* = 0.009) were independent predictors of PE development [39]. Those parameters appear largely unchanged during a 12-month follow-up in 23 patients treated with a no-stent strategy and dual antiplatelet therapy for 1 year after the index ACS [40], suggesting the persistence of the prothrombotic state due to augmented endothelial sheer stress and potentially a need for prolonged intense antithrombotic treatment. While the presence of PE near bifurcations could serve as a simple explanation of the enhanced endothelial sheer stress, a significant proportion of PE leading to ACS arise from vessel areas away from bifurcations (~30%) based on the OPTICO-ACS study [41]. This could be partially explained by the presence of coronary microevaginations, which could serve as a medium of coronary flow disruption and adverse vascular remodeling [42]. Moreover, in such instances, there is a more pronounced activation of the innate immune system, especially CD14^+^CD16^−^ classical monocytes [42].

A recently proposed prevailing theory regarding PE suggests that it involves multiple hits, including distinct activation of neutrophils dependent on toll-like receptor-2 (TLR2) [43]. This activation is believed to be sustained by an increased local expression of hyaluronidase 2, leading to heightened cleavage of hyaluronic acid at the coronary culprit eroded lesion [43]. Consequently, this results in the intensified release of active MMP9 by neutrophils, exacerbating the detachment of luminal endothelial cells under disturbed flow conditions [43]. Besides the significance of the TLR2 pathway in patients with PE, there is a general exacerbation of neutrophil cytotoxicity towards endothelial cells independent of TLR2, which may contribute to the progression of PE [43]. MMP9 may also play a role in bridging innate and adaptive immunity, potentially triggering a complementary T-cell-mediated response in individuals with PE [43].

## 4. Diagnostic Approach toward Plaque Erosion

**Figure 3:**
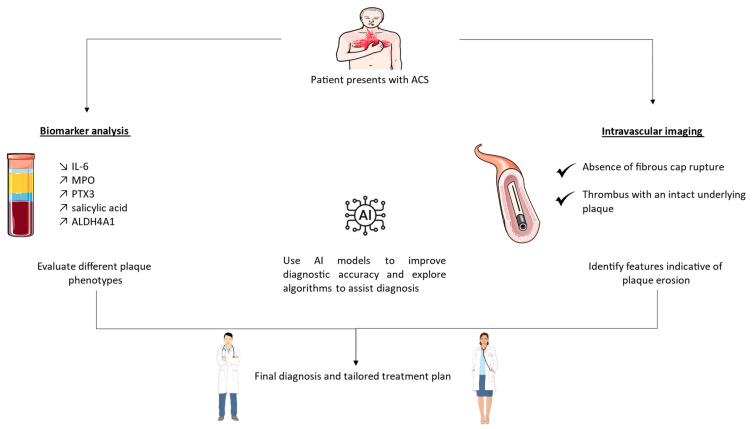
Diagnostic pathway to identify coronary plaque erosion. ACS—acute coronary syndrome, AI—artificial intelligence, IL-6—interleukin-6, MPO—myeloperoxidase, PTX3—pentraxin 3, ALDH4A1—Aldehyde dehydrogenase 4A1.

Understanding the diagnostic approach towards coronary PE necessitates a comprehensive integration of evolving methodologies, such as circulating biomarkers and intravascular imaging techniques, in particular (Figure 3). In this section, we discuss the current advances in the field of PE diagnosis.

### 4.1. Biomarkers

In the field of biomarkers, several research efforts have been made in trying to differentiate between plaque phenotypes. Beginning with inflammatory biomarkers, and according to the OPTICO-ACS study, peripheral blood concentration of IL-6 was significantly lower in patients with PE compared to PR (3.6 ± 3.9 pg/mL vs. 1.4 ± 1.9 pg/mL, *p* = 0.006), but no difference in other common inflammatory biomarkers (CRP, IL-1β) were noted [36]. Importantly, this study found no alterations in inflammatory biomarkers for patients with PE at follow-up examinations [36]. These findings are in line with previous reports showcasing a lower systemic inflammatory burden in PE compared to PR [44]. With regards to CRP, the most widely used circulating inflammatory biomarker, multiple studies have failed to identify differences in its concentration according to plaque phenotype [36,45,46].

Myeloperoxidase (MPO) has been suggested as a systemic biomarker of PE. MPO, serving as an indicator of neutrophil activation, has the potential to promote thrombosis by producing reactive oxygen species, leading to endothelial cell apoptosis and a decrease in nitric oxide levels [47]. Consequently, this exacerbates the deterioration of the endothelial layer in eroded plaques [47]. In a study of 25 consecutive ACS patients (PE: 28%), MPO levels were significantly elevated in PE compared to PR (2500 ng/mL vs. 707 ng/mL, *p* = 0.001) [45]. Similar findings were replicated in a cohort of patients with NSTE-ACS or stable angina, where MPO levels were considerably higher in subjects with PE compared to the other entities (PE: 685.9 ng/mL vs. PR: 340.0 ng/mL vs. stable angina: 272.5 ng/mL, *p* < 0.001) [48]. According to this study, MPO was an independent predictor of PE presence (OR: 1.04, 95% CI: 1.01–1.05, *p* = 0.04) [48]. In the largest prospective study to date in 172 STEMI patients (PE: 46.5%), plasma MPO was higher in PE compared to PR (96.3 ng/mL vs. 41.7 ng/mL, *p* < 0.001), representing an independent predictor (OR: 3.25, 95% CI: 1.37–7.76, *p* = 0.008) but with moderate discriminative ability (area under ROC curve: 0.75, optimal cutoff: 74.2 ng/mL with sensitivity: 64% and specificity: 82%) [49].

Pentraxin 3 (PTX3) is another marker with potentially prognostic significance in the setting of ACS [50]. When considering patients with PE, a high PTX3 concentration (≥6.6 ng/mL) was associated with a higher incidence of the composite endpoint (all-cause death, recurrence of myocardial infarction, stroke, and unplanned revascularization of any coronary artery) during a 1.9-year follow-up (hazard ratio (HR): 5.44, 95% CI: 1.46–20.29, *p* = 0.012) [51]. Based on the study of Zhao et al., PTX3 was associated with fibrous and healing plaques in patients with PE [52]. Prognostically, PTX3 was associated with an over 8-fold increase in the incidence of adverse endpoints in patients with PE, independently of confounders [52].

Apart from inflammation, dyslipidemia is a major mediator of atherosclerosis. While lipid biomarkers, such as Apolipoprotein A-I (ApoA-I) and Apolipoprotein B (ApoB), are strongly associated with the development and progression of atherosclerosis, there is no evidence suggesting their ability to differentiate between PR and PE. A study has previously suggested the presence of plaque instability in cases of high ApoB/A-I ratio [53]. Moreover, given that ApoB/A-I was negatively correlated with fibrous cap thickness [54], we may assume that a lower ratio in the setting of ACS could identify patients with PE. However, this remains to be proven in future studies.

Moving to other less studied markers, in the study of Luo et al. in 123 patients with STEMI, increased salicylic acid was associated with PE presence [55]. However, we should note the mediocre area under the receiver operating characteristic (<0.8) [55]. Given that thrombi in eroded plaques are white, consisting of highly activated platelets, this finding may indicate the existence of disordered salicylic acid metabolism and consequent platelet hyperactivity. Moreover, increased concentrations of salicylic could identify patients with PE who could benefit from intensified antiplatelet therapy regimens, a concept which needs to be further tested.

MicroRNAs are another category of molecules with diagnostic and therapeutic potential in cardiovascular diseases [56,57,58]. Concerning plaque phenotyping, however, evidence remains scarce. Dong et al. studied 76 culprit lesions (32.6% PE) in STEMI patients and in a subanalysis of 16 patients with PE or RP, they found dysregulated expression of 34 microRNAs [59]. Among them, only a higher expression of circulating microRNA-3667-3p remained predictive of PE even after multivariable adjustment (OR: 2.28, 95% CI 1.19–4.35, *p* = 0.01) [59]. Li et al. examined a pool of 24 differentially expressed microRNAs, which was subsequently narrowed to 5 based on the results of the validation cohort [60]. In a replication cohort, the investigators noted lower expression of microRNAs-744-3p, -324-3p, -330-3p, -641, and -4435 in PE compared to PR [60].

Finally, a potential role of Aldehyde dehydrogenase 4A1 (ALDH4A1) was suggested by the study of Li et al. in 312 patients with STEMI (151 with PE) [61]. The circulating plasma ALDH4A1 was significantly increased in subjects with PE compared to PR (4.6 ng/mL vs. 3.5 ng/mL *p* = 0.005), while its abundance in PE was confirmed after immunofluorescence in aspirated coronary thrombus samples [61].

### 4.2. Intravascular Imaging

#### 4.2.1. Optical Coherence Tomography

**Figure 4:**
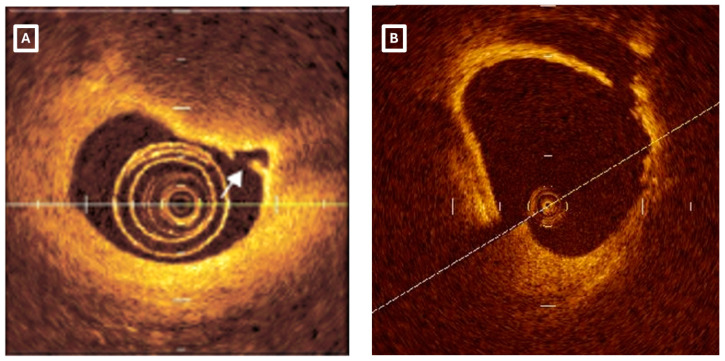
Optical coherence tomography of (**A**) plaque rupture (white arrow) and (**B**) and plaque erosion.

Intravascular imaging is perhaps the method of choice in identifying PE, namely with OCT, as it has a 10-fold higher spatial resolution than IVUS. OCT is unable to view the endothelium monolayer directly. Because endothelial cell loss is a pathological characteristic of PE, this constraint prevents the use of endothelial cell absence as an OCT criterion for identifying PE. OCT has developed a plaque classification algorithm as a result of this. In patients with ACS, the absence of a fibrous cap rupture at the culprit lesion on OCT implies PE, according to this strategy (Figure 4). Identification of a thrombus with an intact underlying plaque is required for a definitive diagnosis of PE. If the luminal surface of the plaque appears sloppy in the absence of a thrombus or if there is a thrombus that hinders the visibility of an underlying plaque without a subjacent lipid accumulation or calcium proximal or distal to the lesion, PE is suspected. This exclusionary approach leads to significant discrepancies in the reported prevalence of PE across various OCT studies. To this end, artificial intelligence may have the potential to improve diagnostic accuracy. Park et al. tested two different deep learning algorithms, convolutional neural networks and transformers, regarding their diagnostic accuracy for PE [62]. They found that the transformer model had slightly superior accuracy at the lesion level (sensitivity: 91.9%, specificity: 81.6%) [62]. When compared to human diagnosticians, the model was superior to less experienced operators but inferior to more experienced operators [62]. Importantly, when it was supplied to less experienced operators, it could assist in improving their diagnostic capabilities toward PE and ultimately outperform the model [62]. Therefore, artificial intelligence could be of critical assistance to flatten out the learning curve in this field.

Concerning prognostic features, an increased abundance of lipids proximally to the erosion site was independently related to a higher incidence of major adverse cardiovascular (HR: 3.74, 95% CI: 1.22–11.5, *p* = 0.02) after a median follow-up of 2.97 years in a cohort of 152 STEMI patients with PE [63]. Proximal lipid content above the median value was associated with a higher fibrous cap thickness, thin cap fibroatheroma prevalence, and the presence of macrophages [63].

#### 4.2.2. Intravascular Ultrasound-Near-Infrared Spectroscopy

High-definition intravascular ultrasound (IVUS) enables detailed examination of the luminal surface and holds potential for detecting PE. Several case series highlight the usefulness of high-definition IVUS in assessing PE [64]. The absence of significant vessel wall abnormalities alongside minor intimal irregularities, with or without thrombus, may indicate PE. Additionally, the presence of fibrotic or lipid plaques exhibiting surface irregularities or layered images, without evidence of cap rupture, could suggest PE. IVUS can also demonstrate the negative vascular remodeling process in the cases of PE compared to the positive remodeling in PR, a finding which is also indicative of the diagnosis [65]. Nonetheless, it is important to acknowledge the inherent difficulty in diagnosing PE using IVUS, since it lacks the resolution necessary for directly visualizing fibrous cap thickness. However, IVUS can be particularly beneficial in cases where PE is suspected and OCT cannot be performed, such as patients with significant renal dysfunction at risk of contrast-associated acute kidney injury. The addition of virtual histology may further aid in characterizing the tissue underneath the plaque by identifying white thrombus and fibrosis [65].

Near-infrared spectroscopy (NIRS), when added to IVUS (IVUS-NIRS), has the ability to quantify lipid core burdens through the lipid core burden index (LCBI). IVUS-NIRS has recently been assessed in 244 patients with ACS regarding its ability to discriminate between PR, PE, and calcified nodule, with an OCT diagnosis as the reference standard [66]. Based on their analysis with a development cohort and a validation cohort, the investigators proposed a three-step approach regarding plaque phenotyping with IVUS-NIRS [66]. Initially, the presence of convex calcium signifies the presence of a calcified nodule (sensitivity 100%, specificity: 99%) [66]. Next, the occurrence of plaque cavitation is a distinct characteristic of definite PR (sensitivity: 97%, specificity: 96%) [66]. In its absence, the maximal LCBI at 4 mm should be assessed, with values below 426 indicating PE (sensitivity: 93%, specificity: 99%, positive predictive value: 93%, negative predictive value 99%) [66]. 

### 4.3. Non-Invasive Imaging

Coronary computed tomography angiography (CCTA) has recently gained attention as a method for characterizing the plaque phenotype of patients with ACS. While the features of vulnerable coronary atherosclerotic plaques that are prone to rupture have been well-established in previous studies [1], its role in identifying PE has not been thoroughly investigated. PE may exhibit lower coronary artery inflammation as assessed by the CCTA-derived pericoronary adipose tissue (PCAT) attenuation, which is considerably higher in cases of PR [67]. Moreover, a recently published study suggested that the absence of vulnerable plaque features (low attenuation, napkin-ring sign, PCAT attenuation in culprit vessel >−70.3) and a zero coronary artery calcium score are independent predictors of PE presence, with an increasing frequency when multiple criteria were fulfilled [68]. In another study, total plaque volume was another variable that related to the presence of PE (OR: 0.995, 95% CI: 0.99–1, *p* = 0.038), with values ≤116 mm^3^ determined as the optimal cutoff.

## 5. Treatment of Plaque Erosion: Contemporary Practice and Future Considerations

As previously stated, patients with PE often have a better prognosis and a lower risk of adverse cardiovascular events compared to those with PR. Since clinical studies showed favorable outcomes in ACS patients with PE versus rupture and thrombi associated with eroded plaques are platelet-rich, it is plausible that a less invasive management strategy (antithrombotic therapy without stenting) may be safe and effective in these patients. In the EROSION trial, 60 ACS patients with PE diagnosed by OCT (96.7% with STEMI), residual diameter stenosis <70%, and thrombolysis in myocardial infarction flow grade 3 on angiography were treated with antithrombotic therapy alone without stent implantation [69]. The use of a glycoprotein IIb/IIIa inhibitor (tirofiban) and manual aspiration thrombectomy was significant (63% and 85%, respectively). After one month, all patients had a repeat coronary angiography and OCT. At 1 month, 78% of these patients attained the main goal of a >50% decrease in thrombus volume. A one-year follow-up study revealed a substantial decrease in median thrombus volume from one month to one year, with 92.5% of patients free of serious adverse cardiovascular events [70]. These findings implied that antithrombotic treatment without stenting may be an option for selected individuals with ACS caused by PE. However, according to the EROSION study’s 4-year follow-up analysis, the incidence of MACE increased to 23%, primarily due to nonurgent target lesion revascularization that was not associated with death, heart failure, stroke, recurrent myocardial infarction, unstable angina-induced rehospitalization, or coronary artery bypass grafting [71]. In an observational analysis of 232 patients, including a fraction from the initial EROSION trial, Yin and colleagues expanded our understanding of this field [72]. During a median follow-up of 2.9 years, 50 of 232 patients (21.6%) developed MACE (6 patients died, 3 patients had nonfatal reinfarction, 29 patients had target lesion revascularization, 36 patients required rehospitalization for angina pectoris, 2 patients had severe bleeding, and 5 patients had a stroke). MACE was predicted by age, percentage of area stenosis, and thrombus load, according to multivariate Cox regression analysis. The threshold values for predictors were age ≥60 years, percentage of area stenosis ≥63.5%, and thrombus load ≥18.5%, respectively, and when all three were present, the risk of MACE spiked to a very high level of 58%. Despite the progress made with the investigation of a no-stent approach in this subset of patients with ACS, large-scale randomized clinical trials are required before recommendations can be made for a routine conservative management with intensive antiplatelet therapy of patients with ACS and PE as the underlying mechanism.

Finally, another open question is whether drug-coated balloon (DCB) angioplasty, might be effective for PE; such an approach might still avoid a stent-based strategy for the majority of patients, but provide a lower area of stenosis, a driver of future MACE. In a retrospective study of consecutive ACS patients with OCT-guided DCB management, target lesion failure was the lowest in the subset of patients with confirmed PE at 3 years [73]. Critically, plaque phenotype (PE) was an independent predictor of target lesion failure [73]. Another observation of this study concerned the post-PCI thrombus burden, with PE and PR having similar target lesion failure rates in cases of residual thrombus burden below 8.4% [73].

Despite their significance in cardiovascular events, ongoing studies on tailored, personalized management of coronary plaque erosions remain sparse. This gap impedes the optimization of treatment strategies, potentially limiting advancements in preventive care and therapeutic interventions for individuals at risk of plaque-related complications. Consequently, universal stenting persists as the standard of care, owing to the absence of additional robust data supporting alternative approaches. However, it should be noted that such approaches may not be adequate in PE, since the findings of a previous study revealed that stents placed on erosion sites exhibit inferior healing, with increased uncovered struts observed at the 6-month mark [74]. These results imply that underlying plaque morphology influences post-stenting vascular response, impacting neointimal coverage and potentially necessitating personalized antiplatelet therapy durations.

## 6. Conclusions and Future Directions

In conclusion, PE has emerged as a significant etiology of ACS, challenging previous assumptions about its prevalence and demographics. With distinct epidemiological features and seasonal variations, PE often affects younger individuals, particularly women, and presents as NSTEMI. Molecular mechanisms highlight reduced inflammation and endothelial shear stress as key factors, while advancements in intravascular imaging offer unprecedented insights into plaque morphology. Artificial intelligence shows promise in enhancing diagnostic accuracy. Therapeutically, less invasive approaches such as antithrombotic therapy without stenting are being explored, alongside the potential of drug-coated balloons to reduce thrombus burden.

The future directions in the diagnosis and treatment of coronary PE are likely to be shaped by advancements in technology, a deeper understanding of molecular mechanisms, and innovative therapeutic strategies. Concerning their diagnosis, continued refinement of intravascular imaging modalities (OCT, NIRS) is expected to enhance the detection and characterization of PE with higher resolution and specificity. Moreover, the determination of specific biomarkers associated with PE for non-invasive diagnosis through blood tests may offer a more accessible and less invasive approach for risk stratification and monitoring. Integration of multiple imaging modalities, including molecular imaging and functional imaging, along with blood-based biomarkers could provide a comprehensive assessment of plaque composition, inflammation, and vulnerability. 

The importance of artificial intelligence should not be overlooked. Machine learning (ML) and artificial intelligence-based methods offer promising avenues for biomarker discovery and validations in coronary PE by leveraging diverse data sources, including genomic, proteomic, imaging, and clinical data. ML algorithms excel in integrating multi-omics data, including genomics, transcriptomics, proteomics, and metabolomics, to capture the complex molecular signatures associated with coronary PE. By analyzing these integrated datasets, ML models can identify patterns and interactions among various molecular features, facilitating the discovery of novel biomarkers. Rigorous validation is essential to ensure the reliability and generalizability of ML-based biomarkers for coronary PE. Cross-validation within training datasets and external validation using independent cohorts are critical steps to assess the robustness and reproducibility of identified biomarkers.

ML algorithms can analyze intravascular imaging data to identify subtle features indicative of PE, such as superficial irregularities or thrombus formation, aiding in the identification of imaging-based biomarkers. Deep learning techniques, particularly convolutional neural networks, have demonstrated remarkable capabilities in analyzing medical images for diagnostic purposes. By training on large datasets of coronary imaging scans, convolutional neural networks can learn complex patterns associated with PE, enabling automated detection and characterization of erosive plaques from imaging data. ML techniques can also analyze electronic health records and clinical databases to extract relevant features associated with coronary PE, such as patient demographics, medical history, laboratory results, and medication usage. By identifying patterns and associations within these clinical datasets, ML models can uncover potential biomarkers and risk factors for PE.

## Abbreviations

ACS	acute coronary syndrome
PR	plaque rupture
PCI	percutaneous coronary intervention
PE	plaque erosion
MMP	matrix metalloproteinase
IL-6	interleukin-6
TNF-α	tumor necrosis factor-alpha
CRP	C reactive protein
VSMC	vascular smooth muscle cell
PCSK9	proprotein convertase subtilisin/kexin type 9
OCT	optical coherence tomography
IVUS	intravascular ultrasound
OR	odds ratio
LAD	left anterior descending
TLR2	toll-like receptor-2
MPO	myeloperoxidase
PTX3	pentraxin-3
HR	hazard ratio
ALDH4A1	aldehyde dehydrogenase 4A1
NIRS	near-infrared spectroscopy
LCBI	lipid core burden index
CCTA	coronary computed tomography angiography
PCAT	pericoronary adipose tissue
DCB	drug-coated balloon
ML	machine learning
